# Localization of Microvascular Changes in Systemic Disease Without Retinopathy Using Optical Coherence Tomography Angiography (OCTA)

**DOI:** 10.3390/jcm14020372

**Published:** 2025-01-09

**Authors:** Alex Hattenhauer, Kimberly Cabrera, Elyana V. T. Locatelli, Pragnya Rao Donthineni, Raquel Goldhardt, Jianhua Wang, Anat Galor

**Affiliations:** 1 Dr. Kiran C Patel College of Allopathic Medicine, Nova Southeastern University, Fort Lauderdale, FL 33314, USA; ah2961@mynsu.nova.edu; 2 Surgical Services, Miami Veterans Healthcare System, Miami, FL 33125, USA; kimberly.cabrera2@va.gov (K.C.); evl17@med.miami.edu (E.V.T.L.); rgoldhardt@med.miami.edu (R.G.); 3 Bascom Palmer Eye Institute, University of Miami Health System, Miami, FL 33136, USAjwang3@med.miami.edu (J.W.); 4 Shantilal Shanghvi Cornea Institute, L.V Prasad Eye Institute, Hyderabad 500034, India

**Keywords:** diabetes mellitus, hypertension, fractal dimension, superficial vascular plexus, deep vascular plexus, optical coherence tomography angiography

## Abstract

**Background**: This study aimed to evaluate the location of retinal fractal dimension (FD) abnormalities in individuals with diabetes mellitus (DM) and hypertension (HTN) without retinopathy. **Methods:** The annular zone of 6 mm × 6 mm OCTA images centered on the fovea was partitioned into thin annuli and analyzed using fractal analysis to measure FDs. **Results:** The cohort (*n* = 114) had an average age of 55.7 years, with 87% self-identifying as male; 29% (*n* = 33) had HTN, 8% (*n* = 9) had DM, and 11% (*n* = 12) had both. Individuals with DM showed significantly lower FDs in the C5 partition of the deep vascular plexus (DVP), located 1.77 mm to 2.14 mm from the foveal center compared to controls without DM (1.57 ± 0.003 vs. 1.58 ± 0.006; *p* = 0.014). Those with both DM and HTN exhibited significantly lower FDs in the entire superficial vascular plexus (SVP) annulus (1.84 ± 0.01 vs. 1.85 ± 0.007; *p* = 0.006), as well as the C3 (1.58 ± 0.02 vs. 1.60 ± 0.02; *p* = 0.008), C4 (1.57 ± 0.002 vs. 1.57 ± 0.01; *p* = 0.036), C5 (1.56 ± 0.01 vs. 1.57 ± 0.008; *p* < 0.001), and C6 (1.58 ± 0.01 vs. 1.59 ± 0.008; *p* < 0.001) partitions of the SVP (1.03 mm to 2.50 mm from the foveal center) and the C4 (1.57 ± 0.008 vs. 1.58 ± 0.008; *p* = 0.015) and C5 (1.57 ± 0.01 vs. 1.58 ± 0.006; *p* = 0.012) partitions of the DVP (1.40 mm to 1.77 mm from the foveal center) compared to the controls with neither DM nor HTN. **Conclusions:** While our study examined FDs in a predominantly male veteran population, our findings align with prior studies that reported lower FDs in DM and HTN cohorts. Our study further localizes these microvascular changes, with the most prominent differences occurring at C5 and C6 of the SVP, representing an area between 1.77 and 2.50 mm from the center of the fovea. These data lay the groundwork for developing screening protocols to identify individuals at risk of developing vasculopathies.

## 1. Introduction

Diabetic retinopathy is the number one cause of vision impairment in working-age adults worldwide [1]. Hypertensive retinopathy, while less likely to cause vision loss, is even more prevalent, estimated to impact upwards of 17% of individuals with hypertension, a disease that affects roughly one third of the adult US population [2]. Treatment options, especially in diabetic retinopathy, may slow down or even reverse retinal pathology. But the optimal strategy in both disorders is early diagnosis and prevention of ocular complications [3]. In addition, interest has arisen on using eye findings as a non-invasive biomarker of systemic disease status [4,5,6,7,8].

Diagnosis of mild non-proliferative diabetic retinopathy (NPDR), the earliest stage of disease, is made once at least one microaneurysm is detected [3]. However, early diabetic retinopathy is often asymptomatic, resulting in progression of the disease to stages that are less amenable to treatment [9]. Fortunately, early screening procedures that result in well-timed treatment have been shown to prevent vision loss in individuals with diabetic retinopathy [10]. Hypertensive retinopathy is also diagnosed and classified via fundoscopic examination, with the lowest Keith–Wagner–Barker classification consisting of slight constriction of the retinal arterioles [2]. Similar to diabetic retinopathy, early manifestations of hypertensive retinopathy can be halted and even reversed, but late retinopathy can sometimes result in permanent vision loss [11]. Like with diabetes mellitus (DM), early hypertensive retinopathy is often asymptomatic, emphasizing the need for screening procedures [9,11].

Beyond positively impacting vision, the detection of early ocular pathology in DM and hypertension (HTN) has been shown to impact systemic management. Research has highlighted that the presence of diabetic retinopathy predicts the development of heart disease, with a 2-fold higher risk of a coronary heart disease (CHD) event (hazard ratio [HR] 2.07; 95% confidence interval [CI] 1.38–3.11) and a 3-fold higher risk of a fatal event (HR 3.35; 95% CI 1.40–8.0) in 1524 Type 2 diabetic individuals [12]. In fact, having even one sign of diabetic retinopathy was associated with a 2.5-fold higher risk of developing heart failure compared to not having any retinopathy among 1021 Type 2 diabetic individuals (HR 2.71; 95% CI 1.46–5.05) [13]. Beyond heart disease, renal disease has also been linked to retinopathy, with increased risk of renal dysfunction in those with retinopathy compared to those without retinopathy (odds ratio [OR] 2.0; 95% CI 1.4 to 2.8) in a US longitudinal study of 10,056 individuals aged 45 to 65 [14]. Similarly, the risk of stroke also increased in individuals with retinopathy compared to those without retinopathy (OR 2.58; 95% CI 1.59 to 4.20) in another US longitudinal study of 10,358 men and women aged 51 to 72 years [15].

Based on the above, an important goal in ophthalmology is identifying diabetic and hypertensive retinopathy as early as possible, even before clinically apparent signs of disease are noted. In this regard, we previously examined the relationships between bulbar conjunctival blood flow and disease severity in individuals with DM. We found that a lower conjunctival blood flow rate correlated with cardiovascular risk, as evidenced by a beta coefficient of the blood flow rate for change in a Framingham score of −0.73 (95% CI −1.34–0.13, *p* = 0.02) and discriminated between diabetic individuals with and without end organ complications (*p* ≤ 0.005, area under the curve (AUC) for axial velocity, cross-sectional velocity, and flow: 0.81, 0.79, and 0.81, respectively) [6,7,8]. However, the capture and analysis of functional slit lamp microscopy images are not fully automated, limiting the utility of this technology in clinical practice. Therefore, there is a demand for a modern, expedited screening tool that can easily be administered in clinical practice while offering comparable insight into systemic processes.

Given these needs, interest has risen on examining high-resolution images generated by optical coherence tomography angiography (OCTA) in individuals with DM and HTN [6]. The unique advantage of OCTA is that the technology is available in most clinics, and it can detect microvascular patterns not apparent on a fluorescein angiograph or slit lamp examination [16]. The capture of OCTA images can be performed within minutes and with automated quantification of vasculature, and results can be available immediately. Machine learning has also been proven to be able to accurately identify vascular features of OCTA images [17]. Current research has noted lower average vessel density (VD) in both the superficial vascular plexus (SVP) and deep vascular plexus (DVP) in diabetic subjects without signs of diabetic retinopathy compared to individuals without DM (*p* < 0.001) [18]. Similar findings have been noted in hypertensive patients with lower VD in the SVP (OR 0.02; 95% CI 0–0.64; *p* = 0.03) and DVP (OR 0.03; 95% CI 0–0.41; *p* = 0.01) compared to individuals without hypertension [19]. With respect to eye pathology, OCTA images have been used to identify early choroidal neovascularization (CNV), with a mean sensitivity of 100% and a mean specificity of 98.8% as compared to fluorescein angiography [20]. These data suggest that OCTA imaging can identify both systemic and local vascular abnormalities, highlighting its potential as a screening tool for diseases associated with vasculopathies.

OCTA imaging allows for the extraction of a wide range of measurements, including fractal dimensions (FDs), which are used to quantify the complexity of vascular patterns. Fractal analysis is particularly useful for evaluating the vasculature in fundus images. One advantage of this approach is that, once the images are skeletonized, fractal measurements are not influenced by variations in vessel width. Instead, they capture the complexity of the microvascular network, offering a more nuanced view of the microvasculature than traditional pixel-per-area density measurements, which can be more affected by the presence of larger vessels. In this paper, vessel density measured using standard pixel-per-area methods will continue to be referred to as VD, while our measurements of fractal dimensions will be labeled as FD to avoid confusion between the two distinct approaches. Both VD and FDs are used to quantify microvascular density, but they represent different methods of assessment.

Prior studies have predominantly focused on OCTA findings in individuals with DM and HTN retinopathy. Less information is available on the utility of OCTA imaging in individuals with DM and HTN but without retinopathy. This paper aims to determine whether OCTA assessed vessel densities, as measured by FDs, differ in individuals with DM and HTN but without retinopathy, as compared to the controls without these conditions, examining differences by the depth (superficial vs. deep) and distance from the fovea.

## 2. Materials and Methods

### 2.1. Study Population

We performed a cross-sectional study of 114 veterans who served during the Gulf War era and who were seen in an eye clinic at the Miami Veterans Affairs (VA) Medical Center between November 2018 and July 2023. Inclusion criteria included a normal external anatomy (e.g., eyelids, conjunctiva, and cornea), as well as at least one set of OCTA images obtained at the VA during the aforementioned time frame. Exclusion criteria included the use of any eye drops beyond artificial tears, eye conditions that could impact dry eye testing (e.g., history of glaucoma, retinal surgery, pterygium, and corneal oedema), history of retinopathy, and any medical conditions that would make study procedures difficult (e.g., neurological and mental health disorders). The exclusion criteria were designed to allow for the focus on biomarkers related to systemic disease by removing potential factors that could interfere with or alter the eye test results, such as pre-existing eye disease. Informed consent was obtained from all subjects. Miami VA Institutional Review Board approval was obtained to allow the prospective evaluation of subjects. This study was conducted in accordance with the principles of the Declaration of Helsinki and complied with the requirements of the United States Health Insurance Portability and Accountability Act.

### 2.2. Demographics and Clinical Data

All demographics were obtained through self-report and verified through chart review and included age, sex, race, ethnicity, and medical history. Mental health status was assessed using the Patient Health Questionnaire for depression and the post-traumatic stress disorder (PTSD) Checklist—Military Version (PCL-M) for PTSD. Other information was collected as available, such as HbA1C (glycosylated hemoglobin). Participants’ diabetic and hypertensive statuses were cross-verified with medical records.

### 2.3. OCTA Image Generation

**Figure 1:**
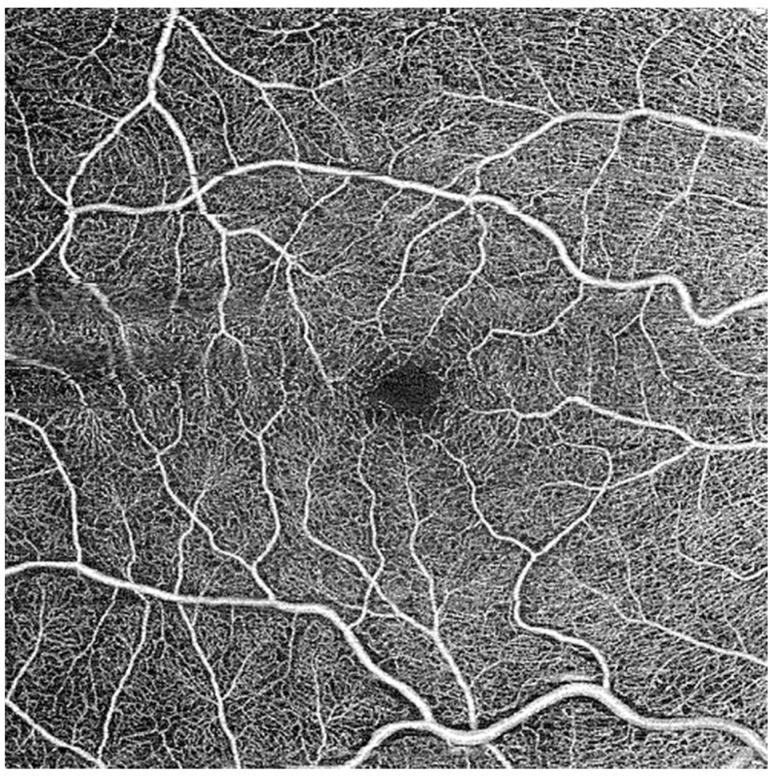
Exported B scan of superficial vascular plexus.

Images were obtained using a Zeiss PLEX Elite 9000 OCTA device (Carl Zeiss Meditec Inc., Dublin, CA, USA). Images of the retinal vasculature are obtained via B scans and analyzed from both intensity and phase information. Each image consisted of a 6 × 6 mm^2^ angiogram, generated in <3 s by taking 4 sequential B scans at a specific y-axis location (Figure S1). The y-axis locations were each scanned during this process. The incorporation of FastTrac technology helped eliminate movement artifacts to generate clear real time retinal imaging. Vasculature from the internal limiting membrane to the inner plexiform layer is termed the superficial vascular plexus (SVP), while vasculature from the inner nuclear layer to the outer plexiform layer is termed the deep vascular plexus (DVP). Angiograms of the SVP and DVP were exported and processed for analysis (Figure 1). Processing of images included separation of large and small vessels and fractal analysis.

### 2.4. Image Processing and Fractal Dimension Determination

**Figure 2:**
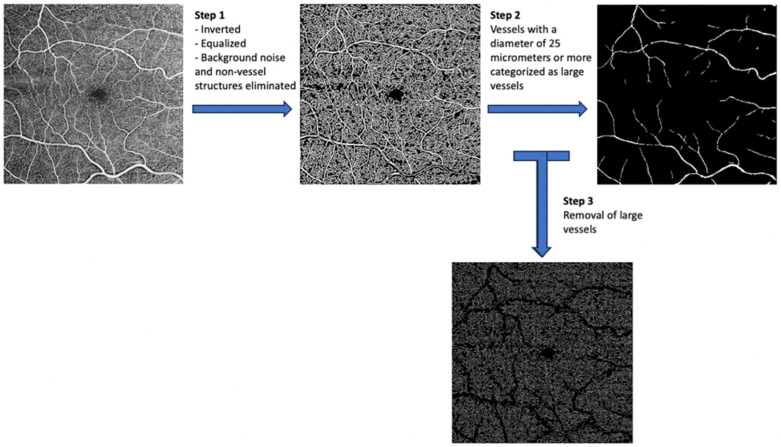
Image processing steps.

Image processing involved several steps, including the separation of large and small vessels, as well as fractal analysis. Initially, binary images were produced using segmentation software within the Matlab environment (R2014). This software inverted the images, equalized them, and eliminated background noise and non-vessel structures (Figure 2). To ensure consistency, left eye images were mirrored to match right eye images before being partitioned and averaged with right eye images.

**Figure 3:**
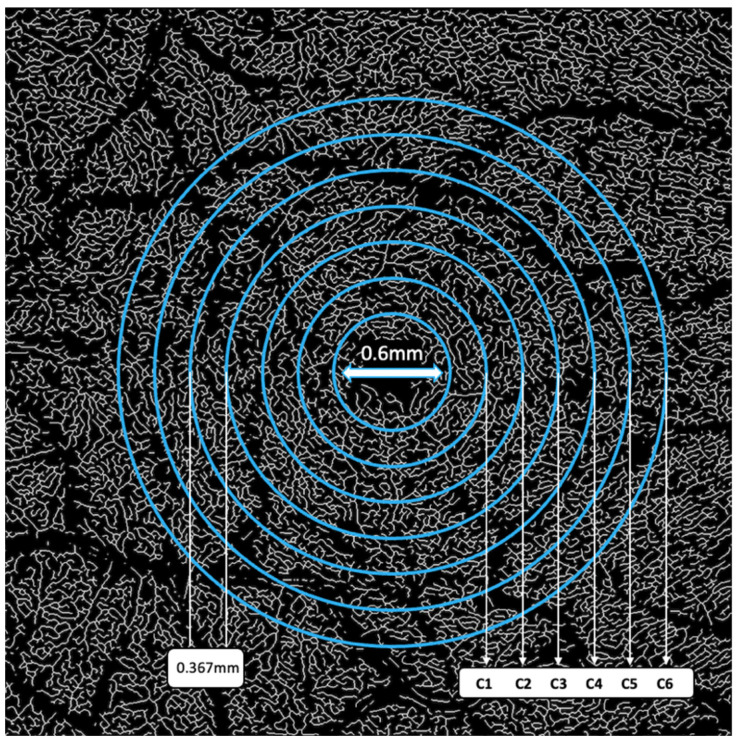
Image partitions.

Vessels with a diameter of 25 μm or more were categorized as large vessels and subsequently removed, leaving behind only microvasculature (Figure 2). The microvessels were then skeletonized and partitioned. The software calculated the center of the foveal avascular zone (FAZ), which was used to define an annular zone in the images. An area with a 0.6 mm diameter, centered on the FAZ, was excised from the image. The remaining area, spanning from 0.6 mm to 5.0 mm in diameter, was defined as the annular zone (entire annulus) with a bandwidth of 2.2 mm. This annular zone was further divided into six annuli, each with a bandwidth of 0.37 mm (Figure 3).

Fractal analysis was performed using a box counting methodology with the assistance of fractal analysis software (TruSoft Benoit Pro 2.0). Default settings included a pixel size of 104 pixels for the largest box used for counting non-empty boxes, with an incremental rotation degree of 15 degrees for searching non-empty boxes. The value obtained from this analysis is the FD.

### 2.5. Statistical Analysis

Statistics were generated through the SPSS 28.0 (SPSS Inc, Chicago, IL, USA) statistical package. Patient and clinical information was summarized and is presented as the mean ± standard deviation (SD). Differences between groups were analyzed through analysis of variance (ANOVA) testing while relationships between variables were distinguished through independent *t*-testing.

## 3. Results

### 3.1. Study Population

The study population consisted of 114 South Florida veterans who met both the inclusion and exclusion criteria with at least one set of OCTA images obtained during the study period. The mean age of the group was 55.7 ± 4.7 years, with most individuals self-identifying as male (86.8%), White (54.4%), and non-Hispanic (64%). A total of 8% (*n* = 9) of individuals had a diagnosis of DM, 29% (*n* = 33) HTN, and 11% (*n* = 12) both DM and HTN (Table 1). Individuals with DM, HTN, and DM and HTN had higher HbA1C values when compared to the controls. Additionally, individuals with DM or HTN had higher rates of hyperlipidemia when compared to the controls. Other co-morbidities were similarly distributed across the groups (Table 1).

### 3.2. Fractal Dimensions

**Figure 4:**
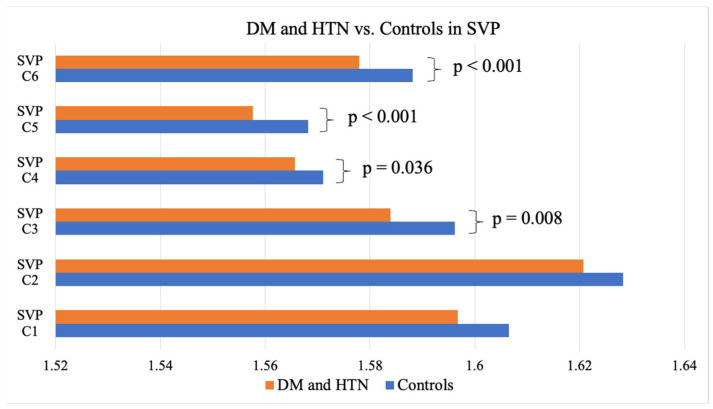
DM and HTN vs. the controls in the SVP. DM, diabetes mellitus; HTN, hypertension; SVP, superficial vascular plexus.

**Figure 5:**
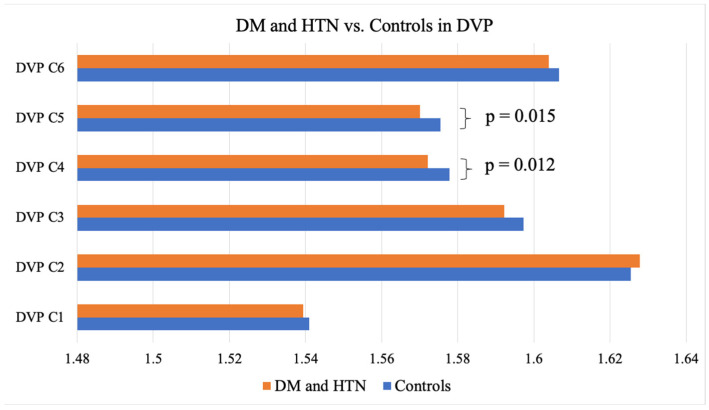
DM and HTN vs. the controls in the DVP. DM, diabetes mellitus; HTN, hypertension; DVP, deep vascular plexus.

As evidenced by the FD means, individuals with DM showed significantly lower FDs in the C5 partition of the DVP, representing an area between 1.77 mm and 2.14 mm away from the center of the fovea when compared to the controls without DM (1.57 ± 0.003 vs. 1.58 ± 0.006; *p* = 0.014) (Table 2). Those presenting with both DM and HTN exhibited significantly lower FDs in the entire SVP anulus (1.84 + 0.01 vs. 1.85 + 0.007; *p* = 0.006), as well as the C3 (1.58 ± 0.02 vs. 1.60 ± 0.02; *p* = 0.008), C4 (1.57 ± 0.002 vs. 1.57 ± 0.01; *p* = 0.036), C5 (1.56 ± 0.01 vs. 1.57 ± 0.008; *p* < 0.001), and C6 (1.58 ± 0.01 vs. 1.59 ± 0.008; *p* < 0.001) partitions of the SVP (1.03 mm to 2.50 mm from the center of the fovea) and the C4 (1.57 ± 0.008 vs. 1.58 ± 0.008; *p* = 0.015) and C5 (1.57 ± 0.01 vs. 1.58 ± 0.006; *p* = 0.012) partitions of the DVP (1.40 mm to 1.77 mm from the center of the fovea) when compared to the controls (Table 2, Figure 4 and Figure 5). Neither the DM, HTN, nor DM and HTN groups showed significant changes in FDs in either the C1 or C2 partitions, representing the areas closest to the foveal avascular zone (0.30 mm to 1.03 mm from the center of the fovea).

### 3.3. Localization and Magnitude of FD Change

**Figure 6:**
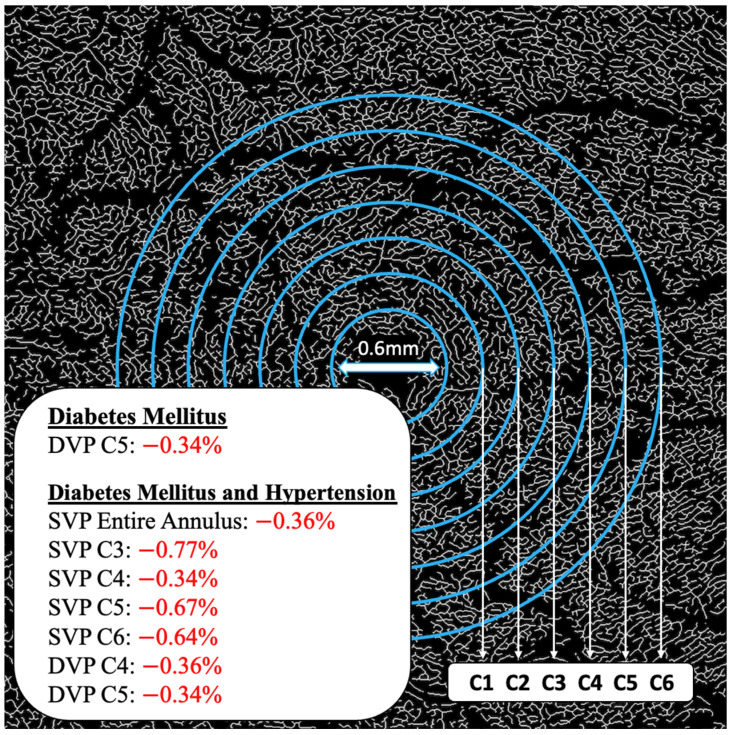
FD differences localized to annulus partitions. DVP, deep vascular plexus; SVP, superficial vascular plexus.

When measuring the entire annulus, individuals with both DM and HTN had 0.36% lower FDs in the SVP than those without DM and HTN. Individuals with DM alone exhibited a 0.34% lower FD measurement in the C5 partition of the DVP compared to the controls without DM. The largest difference (−0.77%) was noted in individuals with both DM and HTN between 1.03 mm and 1.40 mm from the center of the fovea in the SVP (C3) compared to the controls with neither DM nor HTN (Figure 6).

### 3.4. Area Under the ROC Curve

Area under the curve (AUC) measurements showed the highest values when predicting individuals with both DM and HTN, specifically in the C3, C5, and C6 partitions of the SVP, as well as the C4 partition of the DVP (Table 3).

### 3.5. Optimization of the AUC

Optimization of the area under the ROC curve could be achieved by using the sum of the FDs of both the C5 and C6 SVP partitions. Then, using Youden’s index, the value of 3.15 was identified to maximize both the sensitivity and specificity, achieving a sensitivity of 91.7% but a specificity of 60% (Table 4).

### 3.6. HbA1c Correlation

The results of a bivariate analysis of HbA1c values and FD measurements revealed significant negative correlations in the SVP C2 and C5 partitions (−0.195 and −0.223, respectively) as well as the DVP C2, C3, and C4 partitions (−0.189, −0.230, −0.216, respectively) (Table 5).

## 4. Discussion

Our study found that individuals with DM and HTN, but without retinopathy, had significantly lower FD measurements in the SVP between 0.30 mm and 2.50 mm (entire annulus) from the center of the fovea and in the DVP between 1.40 mm and 2.50 mm (C3 to C6) from the center of the fovea on 6 × 6 mm OCTA images when compared to the controls without disease. Specifically, these partitions included C3, C4, C5, and C6 of the SVP and C4 and C5 of the DVP. Combining partitions C5 and C6 (an area between 1.77 and 2.50 mm from the center of the fovea) of the SVP, we could predict the presence of concomitant DM and HTN with an AUC of 0.81 and an optimized Youden’s index of 92% for sensitivity and 60% for specificity. In individuals with only DM and without retinopathy, significantly lower FDs were only observed in the DVP between 1.77 mm and 2.14 mm from the center of the fovea (C5) when compared to the controls. Neither the DM, HTN, nor DM and HTN groups showed significant changes in FDs in either the C1 or C2 partitions, representing the areas closest to the foveal avascular zone (FAZ) (0.30 mm to 1.03 mm from the center of the fovea), suggesting that FD changes may have initially manifested further from the FAZ in individuals with DM and/or HTN without retinopathy. HbA1c values were negatively correlated to FD measurements in the C2 and C5 partitions of the SVP, as well as the C2, C3, and C4 partitions of the DVP, with the strongest correlation occurring in the C3 partition of the DVP, representing an area between 1.03 mm and 1.40 mm from the center of the fovea.

Our study shares similarities and differences with prior studies that examined OCTA findings in DM. Unlike our study, a Japanese study of 29 individuals with Type 2 DM without retinopathy did find lower SVP and DVP VD in a 1.25 mm radius area around the fovea compared to 33 controls without DM (SVP: 44.35% ± 13.31% vs. 51.39% ± 13.05%; *p* = 0.04, and DVP: 31.03% ± 16.33% vs. 41.53% ± 14.08%; *p* < 0.01) [21]. However, this study only examined an area with a 1.25 mm radius around the fovea, limiting its ability to possibly identify more robust changes outside this zone. Similar to our study, others have found VD differences in the area between 1.77 and 2.14 mm, but in a different DM population. A Chinese study compared individuals with proliferative diabetic retinopathy (PDR) (*n* = 25), NPDR (*n* = 43), and DM without retinopathy (*n* = 37) and found a 0.99% lower VD in an area between 1.00 and 3.00 mm from the center of the fovea in eyes with NPDR compared to DM without retinopathy in both the SVP (1.75 + 0.01 vs. 1.73 + 0.02; *p* < 0.01) and DVP (1.75 + 0.01 vs. 1.73 + 0.02; *p* < 0.01) [22]. However, missing from the study was a comparison with a control population without DM.

Our study also shares similarities with prior studies that examined relationships between OCTA metrics and HbA1c. A Spanish study of individuals with Type 1 DM but without retinopathy found that individuals with HbA1c > 7.5% (*n* = 110) had a lower mean VD than individuals with HbA1c 6.5–7.5% (*n* = 94) and HbA1C< 6.5% (*n* = 32) (20.16 ± 1.53 vs. 20.22 ± 1.50 and 20.71 ± 1.97 mm^−1^; *p* < 0.05) [23]. The correlation between FD and HbA1c values in our study align with these findings and localizes the significant negative correlations to the C2 and C5 partitions of the SVP and the C2, C3, and C4 partitions of the DVP. Our results underscore the potential of OCTA imaging in capturing microvascular changes reflective of systemic disease status, particularly in individuals without retinopathy.

In the literature, retinal microvascular changes in HTN share similarities to those found in DM. In a South Korean population with chronic HTN but without retinopathy (45 eyes), SVP VD was lower in the perifoveal area compared to the controls without HTN (50 eyes) (20.5 ± 2.0 vs. 21.7 ± 1.1 mm^−1^; *p* = 0.001) [24]. Interestingly, we did not note significant differences in FD in any annulus partition of both the SVP and DVP in our study. This discrepancy may be attributable to differences in the population demographics or methodology used to measure VD.

While most prior studies reported on the impact of DM or HTN alone, one South Korean study examined the impact of having both DM and HTN on VD. In 91 eyes of cases without retinopathy, a lower VD was noted in an annulus between 1 mm and 3 mm from the foveal center compared to 95 control eyes from individuals without DM or HTN (19.24 + 2.79 vs. 21.81 + 1.15 mm^−1^; *p* < 0.01, combined SVP and DVP measures from en face images) [25]. In our study, we found changes in a similar area away from the foveal center (1.77 and 2.14 mm); however, we localized these changes to the DVP, not the SVP.

While the reasons for our localizing findings are not known, several potential explanations may explain our observations. One hypothesis is that while microvascular damage occurs across the entire retinal vasculature, the increased numbers of endothelial cells in the perifoveal area (corresponding to the higher FD in the area compared to the rest of the retina) release protective factors such as vascular endothelial growth factor (VEGF), which can compensate for the damage locally. This could explain why none of our case groups (DM, HTN, or DM and HTN) had significant changes between 0.3 mm and 1.03 mm from the center of the fovea (C1 and C2). However, significant changes were seen further from the fovea, between 1.03 mm and 2.50 mm, an area with lower FDs and thus potentially less endothelial cells. Another potential explanation relates to the larger vessel diameter outside the fovea. It is hypothesized that subclinical microaneurysm formation may be more common in larger vessels due to higher rates of turbulent flow in those vessels, which would increase the findings of vessel abnormalities and further damage areas further from the fovea. This may explain why the most significant changes in the DM and HTN group were seen in the SVP between 1.77 mm and 2.50 mm from the center of the fovea (C5 and C6), as well as the most significant change in the DM group being in the DVP between 1.77 mm and 2.13 mm from the center of the fovea (C5).

As with all studies, our findings must be considered bearing in mind our study limitations, which included a defined population of predominantly male veterans, with unequal sample sizes across groups, potentially affecting the generalizability of the results. In addition, the small sample size limits our ability to translate these findings into screening protocols or to implement management strategies. Nonetheless, we remain hopeful that future studies will help achieve these goals. Furthermore, our study excluded individuals with DM and HTN retinopathy, so our study design could not examine OCTA findings by retinopathy severity. Furthermore, we lacked information on the DM type in our study population and thus could not comment on potential differences between Types 1 and 2 of the disease. Methodologically, although FDs offer a more precise measurement of microvasculature density, the technique is highly sensitive to subtle changes, potentially detecting changes that are not yet clinically significant. Furthermore, since most prior studies used pixel-per-area measurements to determine vascular density, our ability to compare our values with those of prior studies was limited. A future consideration would be to use a digital enhancement filter to enhance the retinal vasculature [26] to further refine our quantification technique. Finally, potentially confounding variables that were not collected, including diet, exercise, and environmental conditions, may have impacted our results. Despite these limitations, our study provides localization of FD changes in individuals with DM and HTN but without retinopathy and provides insights into the retinal pathology in these individuals. Overall, our findings support the development of retinal imaging technology as a non-invasive biomarker for assessing systemic disease status in DM and HTN. These measurements could be utilized to create screening protocols and inform management strategies. Further studies are needed, however, to replicate our results in more diverse populations and re-examine them in individuals with overt retinopathy. A longitudinal study that would allow for the incorporation of OCTA-based FD analysis in a clinical setting could bolster the clinical importance of this research.

## Figures and Tables

**Table 1:** Population characteristics.

	Controls (*n* = 60)	DM (*n* = 9)	HTN (*n* = 33)	DM and HTN (*n* = 12)	*p*-Value
**Demographics, mean ± SD or % (*n*)**					
Age, years	55.20 ± 4.95	54.56 ± 5.13	56.70 ± 4.23	56.33 ± 4.48	0.41
Sex, male	83% (50)	100% (9)	88% (29)	92% (11)	0.53
Race, Black	30% (18)	56% (5)	52% (17)	58% (7)	0.61
Ethnicity, Hispanic	30% (18)	33% (3)	49% (16)	33% (4)	0.36
**Clinical Characteristics, mean ± SD or % (*n*)**					
HbA1C, %	5.68 ± 0.46 (57/60)	7.32 ± 1.39 ^a,b^ (9/9)	5.98 ± 1.14 ^a^ (32/33)	6.92 ± 0.61 ^a,b^ (12/12)	**<0.001**
Total cholesterol level, mg/dL	193 ± 43.42 (56/60)	168 ± 40.53 (9/9)	186 ± 41.66 (32/33)	160 ± 42.71 (11/12)	0.07
Hyperlipidemia	33% (20)	78% (7) ^a^	58% (19) ^a^	50% (6)	**0.02**
Smoking history Prior Current	17% (10) 15% (9)	11% (1) 22% (2)	24% (8) 18% (6)	25% (3) 17% (2)	0.68
Sleep apnea	55% (33)	67% (6)	67% (22)	58% (7)	0.76
BMI	29.89 ± 4.85 (49/60)	33.47 ± 5.83 (8/9)	32.06 ± 5.21 (27/33)	32.59 ± 6.11 (9/12)	0.11

DM, diabetes mellitus; HTN, hypertension; SD, standard deviation; HbA1C, most recent glycosylated hemoglobin; BMI, body mass index. (#/#), number of patients in the cohort with available data. ^a^ Statistically significant difference compared to the controls. ^b^ Statistically significant difference compared to HTN. **Bold**: statistically significant.

**Table 2:** FDs divided by the presence of DM and HTN.

	Controls (*n* = 60)	DM (*n* = 9)	HTN (*n* = 33)	DM and HTN (*n* = 12)	*p*-Value
**Superficial Vascular Plexus partition (*n*), mean FD ± SD**					
Entire Annulus (114)	1.85 + 0.007	1.85 + 0.007	1.85 + 0.01	1.84 + 0.01 ^a^	0.08
C1 (114)	1.61 ± 0.03	1.61 ± 0.004	1.61 ± 0.005	1.60 ± 0.03	0.68
C2 (114)	1.63 ± 0.02	1.63 ± 0.009	1.62 ± 0.003	1.62 ± 0.02	0.49
C3 (114)	1.60 ± 0.02	1.59 ± 0.006	1.59 ± 0.003	1.58 ± 0.02 ^a^	0.11
C4 (114)	1.57 ± 0.009	1.57 ± 0.005	1.57 ± 0.002	1.57 ± 0.01 ^a^	0.39
C5 (114)	1.57 ± 0.008	1.56 ± 0.006	1.57 ± 0.002	1.56 ± 0.01 ^a^	**0.008**
C6 (114)	1.59 ± 0.008	1.59 ± 0.006	1.59 ± 0.003	1.58 ± 0.01 ^a,b^	0.05
**Deep Vascular Plexus partition (*n*), mean FD ± SD**					
Entire Annulus (114)	1.85 + 0.008	1.85 + 0.008	1.85 + 0.008	1.85 + 0.01	0.67
C1 (114)	1.54 ± 0.05	1.56 ± 0.007	1.53 ± 0.01	1.54 ± 0.07	0.38
C2 (114)	1.63 ± 0.02	1.63 ± 0.004	1.63 ± 0.003	1.63 ± 0.02	0.97
C3 (114)	1.60 ± 0.02	1.59 ± 0.004	1.59 ± 0.002	1.59 ± 0.01	0.42
C4 (114)	1.58 ± 0.008	1.58 ± 0.003	1.58 ± 0.002	1.57 ± 0.008 ^a,b^	0.14
C5 (114)	1.58 ± 0.006	1.57 ± 0.003 ^a^	1.58 ± 0.002	1.57 ± 0.01 ^a^	0.08
C6 (114)	1.61 ± 0.02	1.60 ± 0.008	1.60 ± 0.004	1.60 ± 0.03	0.94

DM, diabetes mellitus; HTN, hypertension; SD, standard deviation. ^a^ Statistically significant difference compared to the controls. ^b^ Statistically significant difference compared to HTN. **Bold**: statistically significant.

**Table 3:** Area under the ROC curve.

Area Under the ROC Curve	DM	HTN	DM and HTN
Superficial Vascular Plexus Entire Annulus	0.411	0.453	**0.703**
Superficial Vascular Plexus C1	0.544	0.532	0.590
Superficial Vascular Plexus C2	0.533	0.600	0.642
Superficial Vascular Plexus C3	0.586	0.642	**0.706**
Superficial Vascular Plexus C4	0.566	0.570	0.669
Superficial Vascular Plexus C5	0.630	0.669	**0.761**
Superficial Vascular Plexus C6	0.663	0.573	**0.767**
Deep Vascular Plexus Entire Annulus	0.561	0.584	**0.593**
Deep Vascular Plexus C1	0.440	0.571	0.494
Deep Vascular Plexus C2	0.488	0.489	0.493
Deep Vascular Plexus C3	0.569	0.560	0.581
Deep Vascular Plexus C4	0.651	0.560	**0.711**
Deep Vascular Plexus C5	0.668	0.558	0.682
Deep Vascular Plexus C6	0.544	0.512	0.558

DM, diabetes mellitus; HTN, hypertension; ROC, receiver operating characteristics. **Bold:** statistically significant.

**Table 4:** Optimized area under the ROC Curve for DM and HTN: Sum of SVP C5 and SVP C6.

**Area Under the ROC Curve**	0.808
**Sensitivity:** For values ≤ 3.15	91.7%
**Specificity:** For values ≤ 3.15	60.0%

ROC, receiver operating characteristics.

**Table 5:** Pearson correlations (r) between FD and HbA1c values.

	HbA1c Pearson Coefficient	*p*-Value
Superficial Vascular Plexus Entire Annulus	−0.127	0.185
Superficial Vascular Plexus C1	−0.161	0.093
Superficial Vascular Plexus C2	−0.195	**0.042**
Superficial Vascular Plexus C3	−0.124	0.198
Superficial Vascular Plexus C4	−0.102	0.288
Superficial Vascular Plexus C5	−0.223	**0.019**
Superficial Vascular Plexus C6	−0.173	0.071
Deep Vascular Plexus Entire Annulus	−0.129	0.180
Deep Vascular Plexus C1	0.062	0.520
Deep Vascular Plexus C2	−0.189	**0.048**
Deep Vascular Plexus C3	−0.230	**0.016**
Deep Vascular Plexus C4	−0.216	**0.024**
Deep Vascular Plexus C5	−0.182	0.057
Deep Vascular Plexus C6	−0.122	0.203

HbA1c, glycosylated hemoglobin. **Bold:** statistically significant.

## Data Availability

The raw data supporting the conclusions of this article will be made available by the authors on request.

## Supplementary Materials

The following supporting information can be downloaded at: https://www.mdpi.com/article/10.3390/jcm14020372/s1, Figure S1: Representative OCT-A and OCT-B scans of Superficial Vascular Plexus.
