# Supplementary figures and images for: Localization of Microvascular Changes in Systemic Disease Without Retinopathy Using Optical Coherence Tomography Angiography (OCTA)
Source: J Clin Med. 2025 Jan 9;14(2):372. doi: 10.3390/jcm14020372 (PMC11765723; doi:10.3390/jcm14020372)

Supplemental Figure 1: Representative OCT-A and OCT-B scans of Superficial Vascular Plexus

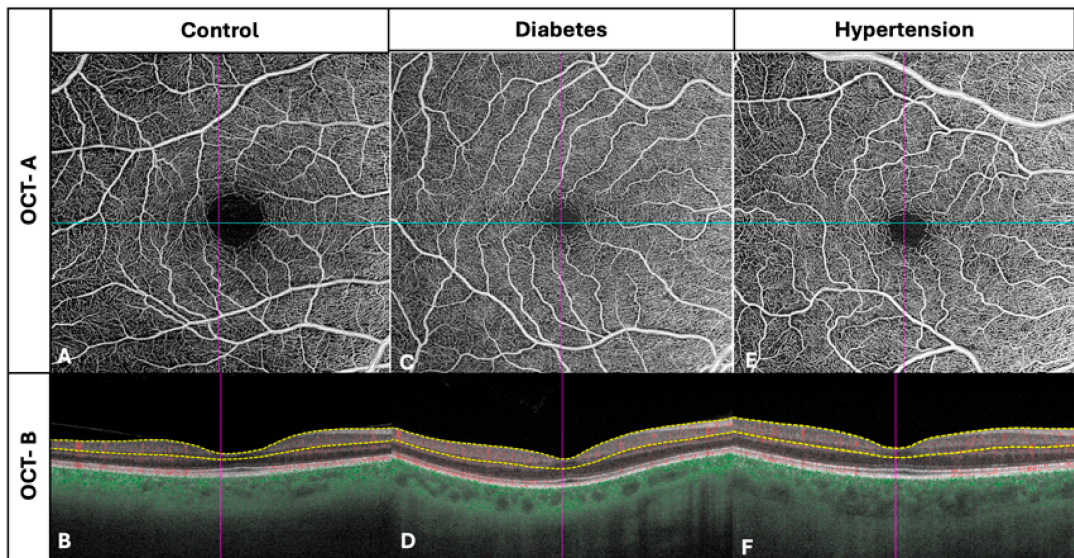

Supplement: Supplementary file 1 [file jcm-14-00372-s001.zip › jcm-3370073-supplementary.pdf]
